# First Human Biomonitoring Evidence of Strobilurin Fungicide Exposure in South China: Impact on Oxidative Stress and Liver Damage

**DOI:** 10.3390/toxics13110908

**Published:** 2025-10-23

**Authors:** Bo Zhang, Shuai Feng, Yanxia Gao, Wenxi Xie, Yiyu Chen, Shiming Song

**Affiliations:** 1 Key Laboratory of Guangdong Higher Education, Institutions of Northeast Guangdong New Functional Materials, School of Chemistry and Environment, Jiaying University, Meizhou 514015, China; 2 School of Environmental Science and Engineering, Sun Yat-sen University, Guangzhou 510275, China; 3 Qiqihar Environmental Monitoring Station, Qiqihar 161005, China; 4 School of Agriculture and Biotechnology, Sun Yat-sen University, Shenzhen 518107, China

**Keywords:** strobilurin fungicides, human exposure, oxidative stress, liver damage

## Abstract

**Background:** Strobilurin fungicides (SFs) are widely detected in the environment, but data on their occurrence in humans and potential health effects are scarce. **Objective:** This study aimed to characterize the exposure to SFs in a human population from South China and to investigate their potential association with biomarkers of oxidative stress and liver damage. **Methods:** In a cross-sectional study, we analyzed serum samples from healthy participants and secondary nonalcoholic fatty liver disease (S-NAFLD) patients. Concentrations of SFs and oxidative stress biomarkers including 8-iso-prostaglandin-F2α (8-PGF_2α_), 11β-prostaglandin F2α (11-PGF_2α_), 15(R)-prostaglandin F2α (15-PGF_2α_), and 8-oxo-7,8-dihydro-20-deoxyguanosine (8-OHdG) were measured. Associations between SF exposure, liver function biomarkers, and S-NAFLD prevalence were assessed using multivariate regression models. A mediation analysis was conducted to explore the role of oxidative stress. **Results:** Azoxystrobin (AZ), fluoxastrobin (FLUO), and fenamidone (FE) were the predominant compounds, with median concentrations ranging from 0.016 to 0.042 ng/mL. Significant positive correlations were observed between all frequently detected SFs and oxidative stress biomarkers (*p* < 0.05). FE was associated with a modest, albeit statistically significant, prevalence of S-NAFLD. AZ and FE were also found to be statistically significantly associated with altered levels of direct bilirubin (DBIL, FDR-q < 0.05). The exploratory mediation analysis indicated a statistically significant indirect effect (17.1% to 31.2%), suggesting that lipid peroxidation biomarkers could serve as potential mediators between AZ exposure and DBIL levels. **Conclusions:** This study provides the first evidence of widespread SF exposure in a South Chinese population and reveals significant associations with oxidative stress and AZ exposure with liver function biomarkers (i.e., DBIL), with exploratory analyses suggesting a potential mediating role of oxidative stress in this relationship. However, the cross-sectional design precludes causal inference, and the modest effect sizes warrant cautious interpretation. These findings highlight the need for further longitudinal research to confirm the hepatotoxicity of SFs in humans.

## 1. Introduction

Strobilurin fungicides (SFs) are a class of widely utilized fungicides in agriculture to control fungal infection, which accounted for about 20% of the global market in 2016 [1]. Among commercially available SFs, azoxystrobin (AZ) is the most prevalent, followed by pyraclostrobin (PYR) [2]. The United States Geological Survey reported that the consumption volume of these two fungicides exceeded 2.5 million pounds and 220 million pounds in the United States and China, respectively [3]. Other marketed SFs include fenamidone (FE), fluoxastrobin (FLUO) orysastrobin (ORY), picoxystrobin (PICO), and trifloxystrobin (TRIF) [4]. Emerging evidence reveals that SFs have been extensively detected in environmental matrices such as water, soil, and dust [5,6,7].

SFs function as potent inhibitors of mitochondrial respiration through specific binding to Q_O_ sites of the cytochrome *bc*_1_ complex in target fungi [8]. However, growing evidence suggests that SFs also induce adverse effects in non-target organisms [9,10]. Exposure to SFs can trigger oxidative stress, leading to the generation of reactive oxygen species (ROS). For example, PYR, TRIF, and PICO have been associated with increased malondialdehyde (MDA) and altered catalase (CAT) and superoxide dismutase (SOD) activity in zebrafish embryos, indicating ROS-induced cell damage [11]. Beyond direct damage, oxidative stress can further generate and reinforce adverse effects such as immunotoxicity [4]. The identification and quantification of related biomarkers provides vital information on the extent of oxidative stress and benefits the diagnosis and prevention of xenobiotic-induced disease [12]. Nevertheless, the relationship between SF exposure and oxidative stress in humans currently remains unknown.

Recent studies have prompted extensive investigation of SFs regarding their potential adverse effects on organisms [13,14,15,16]. Animal studies, for example, have demonstrated significant aquatic toxicity of SFs [15]. The liver, being rich in mitochondria, is particularly vulnerable to SF-induced oxidative stress. Previous studies have provided evidence on liver toxicity of SFs in mammals. Serim et al. reported that PYR caused histopathological alterations and increased apoptotic, proinflammatory, and CYP2E1 mRNA expression levels in rat liver tissues [17]. Nonalcoholic fatty liver disease (NAFLD) is a prevalent liver disorder which is classified into primary and secondary NAFLD (S-NAFLD) [18]. The pathogenesis of S-NAFLD involves multiple determinants, with exposure to environmental chemicals being recognized as a contributing factor to its development and progression [13,19,20]. Evidence has shown that the global prevalence of NAFLD was about 25%, making it the most common cause of chronic liver disease [21]. In China, the incidence of NAFLD is rising significantly, with projections estimating up to 314.58 million cases by 2030 [22]. Given that exposure to SFs has caused liver damage and toxicity in mammals, there is an urgent need to elucidate the potential impact of SFs on the liver and their association with S-NAFLD, particularly in the Chinese population.

In the present study, SFs were firstly detected in serum samples collected from healthy participants and participants with S-NAFLD living in South China. The associations between serum SF level and representative oxidative stress biomarkers, including 8-iso-prostaglandin-F2α (8-PGF_2α_), 11β-prostaglandin F2α (11-PGF_2α_), 15(R)-prostaglandin F2α (15-PGF_2α_), and 8-oxo-7,8 -dihydro-20-deoxyguanosine (8-OHdG) [23,24,25], were analyzed to explore whether SFs triggered oxidative stress in the human body. Moreover, the relationship between serum SF and liver function indicator levels, as well as the potential pathophysiological mediation effects, were investigated in the S-NAFLD group to assess the impact of SF exposure on liver damage.

## 2. Materials and Methods

### 2.1. Chemicals and Reagents

Six target SFs and two metabolites were analyzed in this study, namely, azoxystrobin (AZ), fenamidone (FE), fluoxastrobin (FLUO), orysastrobin (ORY), picoxystrobin (PICO), trifloxystrobin (TRIF), azoxystrobin acid (AZ-acid), and trifloxystrobin acid (TRIF-acid). In addition, 8-PGF2α, 11-PGF2α, 15-PGF2α, and 8-OHdG were measured as biomarkers of oxidative stress in this study. The standards of these compounds (>97% purity) were purchased from AccuStandard (New Haven, CT, USA), J&K Scientific (Beijing, China), and Dr. Ehrenstorfer (Augsburg, Germany). The internal standards, namely d4-8-PGF2α, 13C-8-OHdG, and d5-AZ, were obtained from AccuStandard (New Haven, CT, USA) and Tokyo Chemical Industry (Tokyo, Japan). β-glucuronidase and formic acid were obtained from Sigma-Aldrich (St. Louis, MO, USA). The HPLC-grade ethyl acetate, methanol, and acetonitrile were provided by Merck (Darmstadt, Germany). Moreover, the ultrapure water was prepared by a Milli-Q system (Bedford, MA, USA).

### 2.2. Study Population and Sample Collection

A total of 281 individuals were recruited from hospitals in Zhuhai, Guangdong province, between December 2021 and August 2022. Inclusion criteria for this study were individuals diagnosed as healthy or with S-NAFLD by professional doctors according to clinical diagnostic standards, with no other underlying liver diseases such as viral hepatitis, autoimmune liver disease, congenital diseases, or alcoholic fatty liver disease [26,27]. Diagnosis of S-NAFLD in China followed a sequential exclusion procedure [28]: Suspected fatty liver was first identified via medical imaging and serum biomarkers. Patients with alcohol-induced fatty liver disease were excluded based on defined consumption thresholds (male ≥ 40 g/d, female ≥ 20 g/d for >5 years, or >80 g/d within 2 weeks) [29]. Primary NAFLD (also called MAFLD) related to metabolic syndrome was ruled out by assessing fasting glucose (<6.1 mmol/L), fasting insulin, and homeostasis model assessment-insulin resistance (HOMA-IR, <2.5). Finally, other secondary causes (including steatogenic drugs, nutritional disorders, viral infections, and hypothyroidism) were excluded via standardized clinical criteria. A total of 138 individuals were considered S-NAFLD patients. The ages of the participants ranged from 20 to 75 years, and all were without occupational exposure to the targeted analytes. A schematic overview of the study cohorts is provided in Figure S1. Detailed information on the participants is presented in Table 1. Informed written consent was obtained for each individual before initiation of the study. The experiments in this study were performed in compliance with the Declaration of Helsinki and “The Regulations of Ethical Reviews of Biomedical Research Involving Human Subjects” issued by the National Health and Family Planning Commission of the People’s Republic of China. This study was approved by the Committee on Ethics of Nankai University (No. NKUIRB2023161).

The blood samples were drawn into vacutainer blood collection tubes without any anticoagulants and then centrifuged at 2000 rpm at 4 °C for 10 min to collect serum. Selected hepatic biochemical indexes of the participants were quantified to estimate potential correlations between SF exposure and hepatic injury. All serum samples were stored at −80 °C until required for further analysis.

### 2.3. Sample Extraction and Instrumental Analysis

Detailed information on sample extraction and instrumental analysis are given in the Supporting Information.

Serum samples were extracted using a liquid–liquid extraction (LLE) method reported in our previous studies, with some modifications [30,31]. The concentrations of SFs were measured using an Agilent 1290 Infinity II high-performance liquid chromatography system (Agilent Technologies, Santa Clara, CA, USA), coupled with a SCIEX 5500 triple quadrupole mass spectrometer (SCIEX, Framingham, MA, USA) (Table S1).

### 2.4. Quality Assurance and Quality Control

Instrumental blanks (methanol injection) and procedural blanks (Milli-Q water passed through entire procedure) were evaluated for every 30 samples to confirm the potential contamination. Analysis of blanks revealed detectable levels of AZ at a mean concentration of 0.005 ± 0.001 ng/mL. No other target analytes were detected in the blanks. Serum samples were randomly selected and fortified with 5 ng/mL target SFs to examine the recoveries (*n* = 12). Calibration curves were obtained in the 0.01–50 ng/mL concentration range by using standard solutions of target SFs in methanol, and the regression coefficient (*r*^2^) was >0.99. Spiked samples exhibited recoveries of 65–121% for the target SFs, with an intra-day relative standard deviation (RSD, *n* = 4) of 2–12% and an inter-day RSD of 4–18% (*n* = 12). The limit of quantification (LOQ) was defined as ten times the signal-to-noise ratio (S/N), which varied from 0.001 (ORY) to 0.005 ng/mL (FE), while the limit of detection (LOD) was defined as three times the S/N, which ranged from 0.0003 to 0.0015 ng/mL (Table 2).

### 2.5. Statistical Analysis

SPSS (IBM Corporation, version 21.0, Armonk, NY, USA) and GraphPad Prism (GraphPad Software, version 9.0.0, La Jolla, CA, USA) were used for data analyses. Concentrations below the LOQ were imputed as LOQ/2 for the calculation of median values. To facilitate subsequent model analyses, the concentrations of all SFs were log10-transformed.

Spearman correlation coefficients (*r*) were calculated to examine the correlations among the log10-transformed SFs. To explore the associations between serum SF concentrations and S-NAFLD risk, a multiple linear regression model was used for the continuous variable of liver function indicators. For binary outcome variables (positive vs. negative S-NAFLD), a binary logistic regressions model was used to evaluate the odds ratio (OR) of the average exposure to pollutants with a prevalence of S-NAFLD. Given the cross-sectional design, a mediation analysis was conducted as an exploratory investigation to assess the potential mediating role of oxidative stress biomarkers in the relationship between pollutant exposure and liver function indicators associated with S-NAFLD. The total effect, direct effect, and indirect effect were calculated using PROCESS V4.1 developed by Andrew F. Hayes, in which “model 4” was applicated. The proportion of mediation was defined as the ratio of indirect effect to the total effect. All the models mentioned above were adjusted for potential covaries, such as age (continuous), body mass index (BMI, continuous), and gender (categorical). Statistical significance was set at 0.05. The False Discovery Rate (FDR) using the Benjamini–Hochberg procedure was applied to reduce the likelihood of Type I errors in the regression analysis. Associations with an FDR-adjusted *q*-value < 0.05 were considered statistically significant. Unadjusted *p*-values are reported for transparency, but the interpretation of results primarily relies on the FDR-corrected significance.

## 3. Results

### 3.1. Serum Concentrations and Correlations of SFs in Serum

Table 2 presents the concentrations and detection frequencies (DFs) of the measured SFs. Across all participants, AZ, FLUO, and FE were predominant in serum samples, with DFs above 50%. AZ exhibited the highest concentration among all investigated SFs, with a median concentration of 0.042 ng/mL, followed by FE (0.019 ng/mL) and FLUO (0.016 ng/mL). Although the DFs were low, two metabolites (i.e., AZ-acid and TRIF-acid) were also found in 22% and 21% of participants, respectively. AZ showed a high median concentration in the healthy group (0.049 ng/mL), followed by FE (0.016 ng/mL) and FLUO (0.014 ng/mL). Similarly, AZ had the highest median concentration (0.034 ng/mL) in the S-NAFLD group, followed by FE (0.021 ng/mL) and FLUO (0.016 ng/mL).

**Figure 1:**
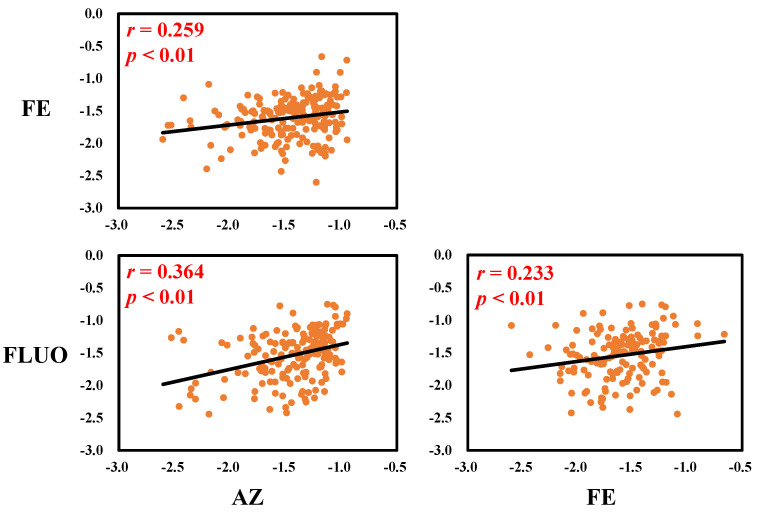
Spearman’s correlation analysis of individual SFs in serum samples collected from all participants. The concentration below the LOQ was excluded from this calculation, and lg-transformed concentrations were used for analysis.

We further conducted correlation analysis of SFs with DFs over 50% among all participants. Pairwise correlation analysis revealed statistically significant positive associations (Spearman’s *r* = 0.233–0.364; *p* < 0.01) among all analyzed SFs within the participants (Figure 1).

### 3.2. Associations Between Serum Concentrations of SFs and Oxidative Stress Biomarkers

**Figure 2:**
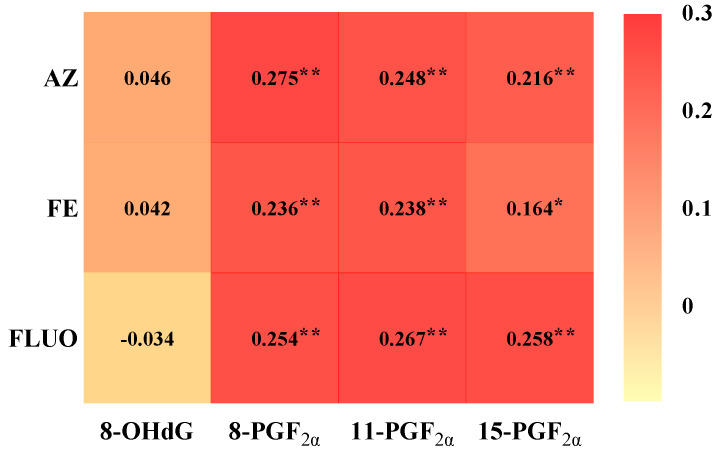
Spearman’s correlation analysis among SFs and oxidative stress biomarkers in serum samples collected from all participants. “*” indicates *p*  <  0.05, and “**” indicates *p*  <  0.01. The concentration below the LOQ was excluded from this calculation, and lg-transformed concentrations were used for analysis.

Oxidative stress has been considered one of the main toxicity effects in animal studies related to SF exposure [11]. Thus, the relationships between concentrations of oxidative stress biomarkers and SFs in serum samples obtained from all participants were investigated to evaluate similar effects in the human body. Figure 2 shows that 8-PGF_2α_, 11-PGF_2α_, and 15-PGF_2α_ were significantly positively correlated with AZ (*r * =  0.216–0.275; *p*  <  0.01), FE (*r * =  0.164–0.238; *p*  <  0.05), and FLUO (*r * =  0.254–0.267; *p*  <  0.01) in serum. Meanwhile, 8-OHdG presented no significant correlation with the investigated SFs, including AZ, FE, and FLUO, in serum samples.

### 3.3. Associations Between SF Exposure and the Occurrence of Liver Damage

**Figure 3:**
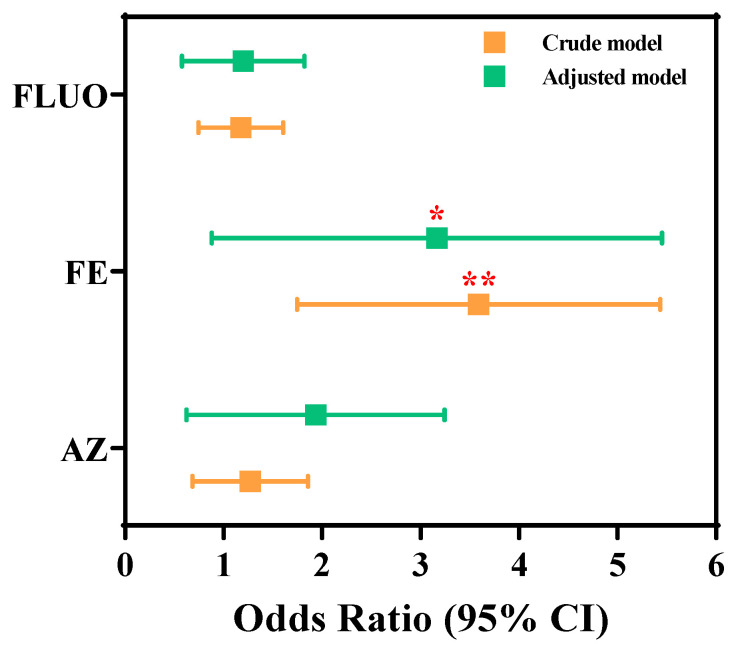
Odds ratios of S-NAFLD prevalence based on serum SF concentrations in this study. Healthy controls are treated as reference; multiple logistic regression models were used out to assess the OR of NAFLD prevalence, which were estimated by 1-standard deviation higher difference in lg-transformed SFs as continuous variables; the model is adjusted for gender (categorical), age (continuous), and BMI (continuous); “*” indicates *p*  <  0.05, and “**” indicates *p*  <  0.01.

Figure 3 presents the odds ratios (ORs) for the prevalence of S-NAFLD, accompanied by 95% CIs for SFs across all participants. Serum FE was significantly and positively associated with S-NAFLD prevalence in both the crude (OR: 3.274; 95% CI: 1.924–5.569; *p*-trend < 0.01) and adjusted (OR: 2.615; 95% CI: 1.205–5.674; *p*-trend < 0.05) model. Furthermore, AZ and FLUO were positively correlated with S-NAFLD prevalence without statistical difference (*p*-trend > 0.05). The OR values were 1.639 (95% CI: 0.798–3.367) and 1.091 (95% CI: 0.637–1.869) in the adjusted model. Thus, the results suggested that serum FE was statistically significant, with an increased prevalence of S-NAFLD in the investigated population.

**Figure 4:**
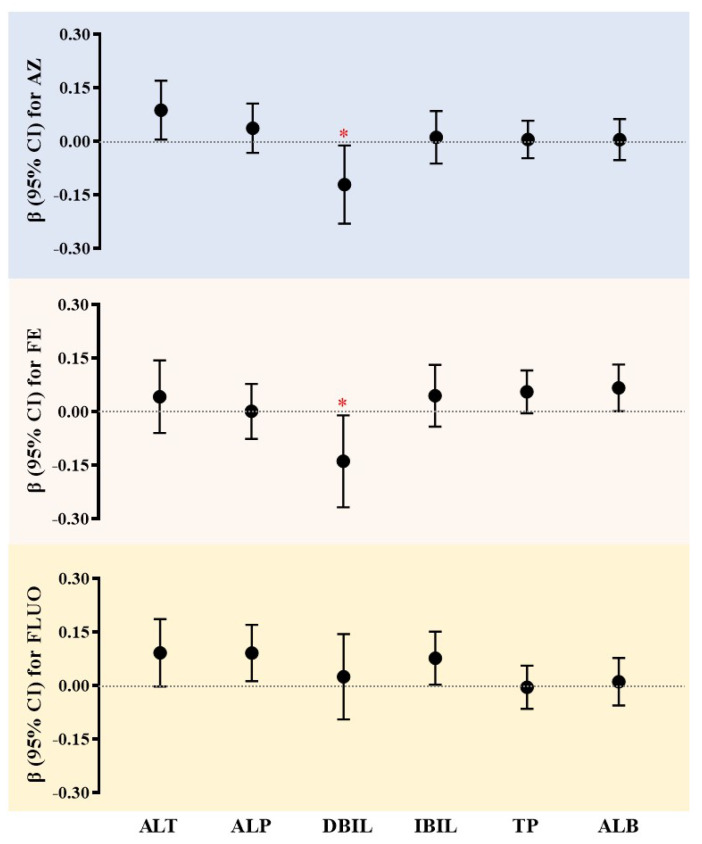
Multiple linear regression coefficients (β, 95%CI) with significant differences among SFs in serum and liver function indicators of S-NAFLD group. ALT: alanine aminotransferase; ALP: alkaline phosphatase; DBIL: direct bilirubin; IBIL: indirect bilirubin; TPs: total proteins; ALB: albumin proteins; “* ” indicates FDR-q  <  0.05; the concentration below LOQ was excluded from this calculation; lg-transformed concentrations were used in this analysis.

Subsequently, we conducted multiple linear regression analyses to evaluate the relationship between AZ, FE, and FLUO with liver function indicators in the S-NAFLD group (Figure 4). After applying FDR correction for multiple testing, the most robust findings that emerged were the associations between AZ and direct bilirubin (DBIL, −0.121; 95% CI: −0.231–−0.012; FDR-*q* < 0.05), as well as FE and DBIL (−0.139; 95% CI: −0.268–−0.010; FDR-*q* < 0.05). Although associations between AZ and alanine aminotransferase (ALT, 0.087; 95% CI: 0.012–0170; *p* < 0.05), FE and albumin (ALB, 0.067; 95% CI: 0.001–0.132; *p* < 0.05), FLUO and alkaline phosphatase (ALP, 0.091; 95% CI: 0.005–0170; *p* < 0.05), and indirect bilirubin (IBIL, 0.077; 95% CI: 0.002–0151; *p* < 0.05) were nominally significant, they should be interpreted with caution, as they did not survive multiple testing correction and may represent false positives or require validation in larger, independent cohorts. Results for aspartate aminotransferase (AST) and gamma-glutamyl transferase (GGT) are presented in Table S3, where no significant associations were found with serum SF concentration. It is suggested SF exposure was associated with a modest, albeit statistically significant, alternation with several indicators, indicating that the absolute changes were within the conventional clinical reference range. It is important to note that the wide confidence interval indicates substantial uncertainty regarding the magnitude and precision of this association.

**Figure 5:**
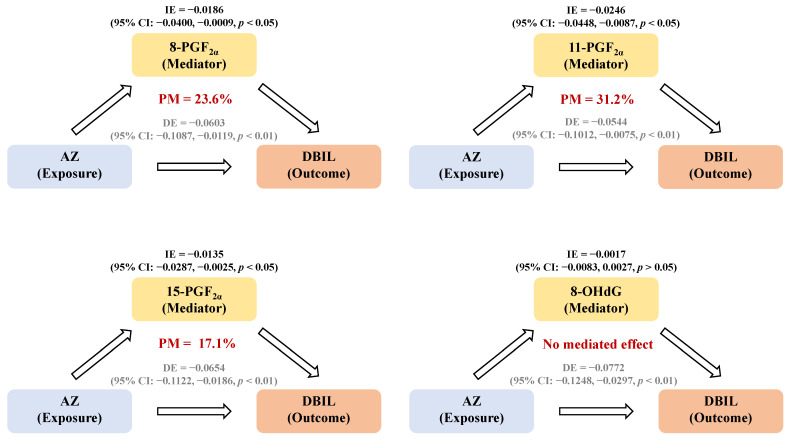
The mediating effects of oxidative stress biomarkers in associations between AZ exposure and DBIL outcomes. The concentration below the LOQ was excluded from this calculation; lg-transformed concentrations were used in this analysis. Contents in red means proportion of mediation. Contents in gray means direct effects.

The association between oxidative stress and pathophysiological processes highlights the potential utility of biomarker identification, thereby paving the way for enhanced disease diagnostic validation. Figure 5 shows the results of an exploratory mediation analysis, which investigated a potential pathway whereby SF exposure might be associated with alternations in liver function indicators through oxidative stress in the S-NAFLD group. The results indicated statistically significant indirect effects of 8-PGF_2α_ (−0.0186; 95% CI = −0.0400–−0.0009; *p*  <  0.05), 11-PGF_2α_ (−0.0246; 95%CI = −0.0448–−0.0087; *p*  <  0.05), and 15-PGF_2α_ (−0.0135; 95% CI = −0.00287–−0.0025; *p*  <  0.05), induced by AZ exposure, which demonstrated potential mediation effects of up to 23.6%, 31.2%, and 17.1%, respectively, in the observed alternation in serum DBIL level. However, we emphasize the preliminary nature of this finding due to the study’s cross-sectional design.

## 4. Discussion

Most SFs were mainly reported in aquatic environments due to their migration after application in agriculture, implying the possible source of human exposure [4]. After application, SFs can enter adjacent water bodies through wet deposition and leaching [32]. Our results suggested that AZ was the predominant SF in both the healthy and S-NAFLD group. Liu et al. detected AZ in drinking water collected in Central China, with an average concentration as 0.001 ng/mL, which was over three magnitudes higher than TRIF and FLUO [33]. Moreover, conventional water treatment processes appear inefficient at removing AZ, with approximately 60% persisting from source water to tap water. In addition, AZ was detectable in dust samples (median concentration: 0.70 ng/g) and wallboard, indicating that direct contact with these matrices may be a key contributor to AZ accumulation in humans [5,34,35]. It is important to note that SFs are never isolated when found within environmental matrices. For example, Zhao et al. observed AZ, FLUO, and TRIF in surface water samples collected from Dongjiang river, southern China. Except for environmental matrices, AZ and FLUO were also simultaneously detectable in fruits and vegetables [36]. FE was also found in agricultural products [37]. These results implied similar exposure sources of SFs. Nevertheless, the measured serum concentrations of SFs in our participants were in the low range. This pattern is highly indicative of chronic, low-level exposure from environmental and dietary sources, rather than acute occupational exposure. Similar low levels of other pesticides have been reported in general populations worldwide, attributed to the ubiquity of these compounds in the environment, including in dust, water, and food, as we mentioned above. The high DFs of AZ and FLUO underscore their widespread exposure, even among participants without known occupational contact. Therefore, our findings raise concerns about the potential population-wide health impacts of SF exposure.

Serum concentrations of ORY, PICO, and TRIF were unexpectedly elevated in healthy controls relative to S-NAFLD patients. This inverse association may reflect disease-altered toxicokinetics. Compromised hepatic function in S-NAFLD patients could prevent the bioactivation of these compounds [38,39], leading to their relative accumulation in healthy individuals. Unmeasured confounding exposure sources or selection bias through the hospital-based design could also be one explanation. Notably, this pattern was compound-specific, contrasting with the opposite trend observed for FE and FLUO. This shows that future studies must account for compound-specific toxicokinetics of SFs.

Interestingly, AZ showed a much higher DF than AZ-acid, while the value of TRIF was even lower than its metabolite (Table 2). Thus, we further calculated concentration ratios using serum samples detected for both metabolites and parent compounds, indicating that the median values were 0.11 and 1.17 for AZ-acid/AZ and TRIF-acid/TRIF, respectively (Figure S2). These results might be attributed to the variation in elimination between AZ- and TRIF-related compounds. Hu et al. reported a much higher DF (70% vs. 10%) and one magnitude higher concentration (0.09 ± 2.53 ng/mL vs. 0.003 ± 0.35 ng/mL) of urinary AZ-acid when compared with AZ in newborns [40]. The authors further conducted animal tests and observed that urinary concentrations of AZ-acid were two magnitudes higher than those of AZ after oral administration [40]. These results suggested that AZ-acid can be easily excreted through urine, which leads to its less occurrence in serum. In contrast, TRIF has a higher octanol–water partition coefficient (log K_OW_) than AZ (4.5 vs. 2.5), which indicates that TRIF is more lipidophilic [1]. TRIF-acid was even more persistent than TRIF [41]. Due to a lack of information on the adverse effects to humans, further studies on the toxicity of these metabolites should be carried out in the future.

Current biomonitoring data on SFs in human populations remain scarce, with urinary concentrations of AZ and AZ-acid concentrations (range: 0.003–3.04 ng/mL) being quantified solely in a United States cohort [40]. However, toxicological studies have demonstrated that SFs had carcinogenic potential, causing liver tumors and malformations in mammals [42,43,44]. Our results suggest significant correlations between SF exposure and 8-PGF_2α_, 11-PGF_2α_, and 15-PGF_2α_, which are the indicators of lipid peroxidation [45]. Although no previous human data exist, multiple studies demonstrated that SF exposure altered normal lipid metabolism in zebrafish and rats by inducing fluctuations in malondialdehyde concentration, indicating the results of lipid peroxidation [46,47,48]. Therefore, the results of the current study imply that SF may serve as one factor influencing oxidation of lipids in the human body. Nevertheless, these findings should be interpreted with caution due to the cross-sectional nature of this study and lack of specificity of F_2_-isoprostanes, which could be influenced by potential unmeasured confounders such as systemic inflammation and diet. Future longitudinal or experimental studies should employ a broader oxidative stress panel, including markers of aldehyde production (e.g., 4-Hydroxynonenal, Malondialdehyde) and redox status (e.g., Glutathione/Glutathione Disulfide ratio), to validate the role of SFs in oxidative stress.

Our study provides evidence of a statistically significant but modest association between SF exposure and liver function indicators. DBIL is a key factor for evaluating bile excretion function and hepatocyte binding ability and is also regarded as a biomarker of liver damage in humans [49]. Given the small effect sizes and the imprecision of the estimates, however, these findings should be interpreted with caution. Our findings observed associations between SF exposure and liver biomarkers, reflecting subtle, subclinical variations in most participants. The changes that we report are below the thresholds typically used to diagnose overt liver disease in a clinical setting. Therefore, the clinical significance for any single individual remains uncertain. However, from a public health and environmental risk assessment perspective, the identification of these exposure-related subclinical shifts is noteworthy. Even minor shifts in the population distribution of liver enzymes, driven by environmental exposures, could potentially increase the number of individuals at the higher end of the normal range, who may be at greater risk of future liver pathology [50]. Ultimately, longitudinal studies are required to determine whether these small, exposure-associated variations predict an increased risk of developing liver dysfunction over time.

Epidemiological research on disorders of liver function induced by SFs is lacking for humans. Several in vivo and animal studies have provided evidence that exposure to AZ and FE induces liver oxidative stress, apoptosis, and hepatopancreas toxicity [51,52,53,54]. Our results implied that 8-PGF_2α_, 11-PGF_2α_, and 15-PGF_2α_ may serve as potential mediators between serum AZ and the altered DBIL value in the S-NAFLD group. Moreover, the role of lipid peroxidation in the progression of NAFLD has been demonstrated through the alleviation of histopathological parameters by antioxidant supplementation. For example, vitamin E treatment decreased NAFLD-associated inflammation in clinical trials [55]. Therefore, our study suggested that the AZ-associated liver function indicator change may involve metabolic disorder via lipid peroxidation, subsequently altering DBIL levels in the human body. However, it should be noted that the cross-sectional design and the modest sample size may limit the statistical power and stability of the mediation model. Therefore, our mediation results should be interpreted not as evidence of a causal pathway, but as the identification of a statistically significant associative pathway that is consistent with a plausible biological mechanism. The complete mechanisms of AZ exposure, including the mediating effect of lipid peroxidation, on S-NAFLD require further investigation, which has substantial implications for disease prevention and potential benefits for public health.

## 5. Strengths and Limitations

This study makes several significant contributions to the field of environmental health, with its core strengths being the following: This is the first study to systematically report internal exposure levels of multiple SFs and their metabolites in a human population, and it moves beyond simple exposure–outcome associations by exploring the potential mediating role of oxidative stress. It delivers pioneering biomonitoring data on SFs in a South Chinese cohort and provides a plausible biological mechanism for the observed hepatotoxicity, linking SF exposure to subclinical liver effects through a pathway supported by the data.

This study also has several limitations: A primary limitation of this study is its cross-sectional design and the recruitment of participants from hospital settings, which may introduce selection bias and affect the generalizability of our findings. Individuals attending hospitals are likely to have different health profiles, greater disease severity, or different health-seeking behaviors compared to the general population. In the context of our study, this could mean that the observed association between SF exposure and liver damage or S-NAFLD might be overestimated if our sample comprised a higher proportion of high-risk patients. Conversely, if individuals with severe comorbidities were excluded, the association might be underestimated. Therefore, while our results are highly relevant for understanding this relationship in clinical populations, caution is warranted when extrapolating them to the community at large. The associations that we observed between SF exposure and liver damage or S-NAFLD should be interpreted as correlational, and no causal inferences can be drawn. Future population-based cohort studies are essential to confirm the prevalence and strength of this association in a more representative sample.

In addition, although we statistically adjusted for these factors in all our multivariate models, the possibility of residual confounding cannot be entirely eliminated. The imperfect matching could have led to an over- or underestimation of the true association if these demographic factors are independently linked to both the exposure and the outcome. Therefore, our results must be interpreted with this caution in mind. Future studies with population-based controls and careful matching on key demographic variables are warranted to confirm our findings.

Finally, although we adjusted for key covariates including age, gender, and BMI in our multivariate analysis, we cannot rule out residual confounding by other unmeasured variables. For instance, dietary patterns (e.g., high fructose intake), levels of physical activity, socioeconomic status, and exposure to other environmental contaminants are known or suspected risk factors for NAFLD and may also be correlated with SF exposure through dietary or other pathways. If present, such confounding factors could have influenced the magnitude and precision of the observed associations. For example, if individuals with healthier lifestyles (e.g., better diet and more exercise) also consume diets that are lower in SF residues, the true association between SF exposure and NAFLD might be even stronger than what we observed. Conversely, if unmeasured factors are positively associated with both exposure and outcome, our estimates might be overinflated. Therefore, our findings should be interpreted as preliminary evidence of an association, and future prospective studies that carefully collect data on these additional potential confounders are necessary to confirm our results and provide more definitive evidence.

## 6. Conclusions

This study provides initial evidence of occurrence of SFs in serum samples collected from residents in China. AZ, FLUO, and FE were the main components in serum samples (DF > 50%). The median concentration of SFs ranged from <LOQ to 0.042 ng/mL across all participants. All frequently detected SFs presented significant positive correlations with oxidative stress biomarkers (i.e., 8-PGF_2α_, 11-PGF_2α_, and 15-PGF_2α_) in all participants, suggesting that SFs may cause oxidative stress by triggering lipid peroxidation in the human body. Thus, exposure to FE was linked to an increased prevalence of S-NAFLD in the investigated participants. AZ and FE showed significant associations with alterations in liver function biomarker (i.e., DBIL), with exploratory analyses suggesting a potential mediating role of oxidative stress in this relationship. To build upon these findings and investigate potential causality, future longitudinal studies or randomized controlled trials are warranted to examine the temporal relationship and the efficacy of interventions targeting SF exposure.

## Figures and Tables

**Table 1:** Basic characteristics and distribution of metabolic and inflammatory indicators of the study participants (*n* = 281).

Characteristics	All Participants	Group	
	(*n* = 281)	Healthy (*n* = 143)	S-NAFLD (*n* = 138)
Age (years)	37.1 ± 11.8	30.0 ± 7.93	44.4 ± 10.6
BMI [kg/m^2^, *n* (%)]			
BMI < 18	10 (3%)	10 (7%)	0 (0%)
18 ≤ BMI < 24	157 (56%)	109 (76%)	48 (35%)
≥24	114 (41%)	24 (17%)	90 (65%)
Gender [*n* (%)]			
Female	134 (48%)	91 (64%)	43 (31%)
Male	147 (52%)	52 (36%)	95 (69%)
ALT (U/L)	36.5 ± 27.9	25.7 ± 7.39	48.5 ± 35.8
AST (U/L)	38.7 ± 17.5	25.6 ± 5.27	53.2 ± 14.5
GGT (U/L)	36.5 ± 23.4	26.1 ± 8.92	47.5 ± 28.3
ALP (U/L)	84.3 ± 28.1	79.0 ± 23.3	89.2 ± 31.8
TBIL (μmol/L)	16.4 ± 6.21	12.9 ± 3.47	20.0 ± 6.42
DBIL (μmol/L)	6.90 ± 3.39	5.64 ± 1.82	8.18 ± 4.12
IBIL (μmol/L)	9.49 ± 3.93	7.28 ± 2.35	11.9 ± 4.01
TP (g/L)	68.9 ± 11.1	72.1 ± 5.47	65.1 ± 14.0
ALB (g/L)	40.1 ± 7.19	42.1 ± 4.61	37.8 ± 8.52
GLB (g/L)	28.7 ± 6.66	30.0 ± 5.15	27.3 ± 7.76

Abbreviations: BMI, body mass index; ALT, alanine aminotransferase; AST, aspartate aminotransferase; GGT, gamma-glutamyl transferase; ALP, alkaline phosphatase; TBIL, total bilirubin; DBIL, direct bilirubin; IBIL, indirect bilirubin; TP, total protein; ALB, albumin; GLB, globulin.

**Table 2:** Concentrations of serum SFs among study participants (ng/mL).

	LOD	LOQ	Range	All (*n* = 281)			Health (*n* = 143)			S-NAFLD (*n* = 138)		
				DF	Median	Geomean	DF	Median	Geomean	DF	Median	Geomean
AZ	0.0012	0.004	<LOQ-0.118	89%	0.042	0.030	83%	0.049	0.028	94%	0.034	0.032
FE	0.0015	0.005	<LOQ-0.218	75%	0.019	0.014	62%	0.016	0.011	88%	0.021	0.018
FLUO	0.0012	0.004	<LOQ-0.178	65%	0.016	0.012	60%	0.014	0.011	70%	0.016	0.012
ORY	0.0003	0.001	<LOQ-0.024	49%	<LOQ	0.002	73%	0.006	0.004	25%	<LOQ	0.0014
PICO	0.0006	0.002	<LOQ-0.075	47%	<LOQ	0.003	58%	0.008	0.005	35%	<LOQ	0.0021
TRIF	0.0009	0.003	<LOQ-0.025	17%	<LOQ	<LOQ	34%	<LOQ	<LOQ	0%	<LOQ	<LOQ
AZ-acid	0.0003	0.001	<LOQ-0.023	22%	<LOQ	<LOQ	36%	<LOQ	<LOQ	7%	<LOQ	<LOQ
TRIF-acid	0.0003	0.001	<LOQ-0.062	21%	<LOQ	<LOQ	38%	<LOQ	<LOQ	3%	<LOQ	<LOQ

Abbreviations: LOD, limit of detection; LOQ, limit of quantification; DF, detection frequency; FLUO, fluoxastrobin; ORY, orysastrobin; AZ, azoxystrobin; PICO, picoxystrobin; FE, fenamidone; TRIF: trifloxystrobin.

## Data Availability

The original contributions presented in this study are included in the article/Supplementary Material.

## Supplementary Materials

The following supporting information can be downloaded at https://www.mdpi.com/article/10.3390/toxics13110908/s1. Supplementary Materials Text: The procedure for sample extraction and instrumental analysis; Table S1: The gradient of mobile phases for SFs; Table S2: The precursor (Q1), product ion (Q3), declustering potential (DP), and collision energy (CE) of targeted antimicrobials and oxidative stress indicator quantification and qualification; Table S3: Associations between serum concentration of SF (DF > 50%) and selected liver function indictors; Figure S1: Schematic diagram of the analytical cohort structure; Figure S2: Concentration ratio of AZ/AZ-acid and TRIF/TRIF-acid in serum samples collected from all participants.

## References

[1] Zhang C., Zhou T., Xu Y., Du Z., Li B., Wang J., Wang J., Zhu L. (2020). Ecotoxicology of Strobilurin Fungicides. Sci. Total Environ..

[2] Mao L., Jia W., Zhang L., Zhang Y., Zhu L., Sial M.U., Jiang H. (2020). Embryonic Development and Oxidative Stress Effects in the Larvae and Adult Fish Livers of Zebrafish (*Danio rerio*) Exposed to the Strobilurin Fungicides, Kresoxim-Methyl and Pyraclostrobin. Sci. Total Environ..

[3] United States Geological Survey (2019). Estimated Annual Agricultural Pesticide Use. https://www.usgs.gov/tools/estimated-annual-agricultural-pesticide-use.

[4] Leite F.G., Sampaio C.F., Pires J.A.C., de Oliveira D.P., Dorta D.J. (2024). Toxicological Impact of Strobilurin Fungicides on Human and Environmental Health: A Literature Review. J. Environ. Sci. Health Part B Pestic. Food Contam. Agric. Wastes.

[5] Cooper E.M., Rushing R., Hoffman K., Phillips A.L., Hammel S.C., Zylka M.J., Stapleton H.M. (2020). Strobilurin Fungicides in House Dust: Is Wallboard a Source?. J. Expo. Sci. Environ. Epidemiol..

[6] Li X.Y., Qin Y.J., Wang Y., Huang T., Zhao Y.H., Wang X.H., Martyniuk C.J., Yan B. (2021). Relative Comparison of Strobilurin Fungicides at Environmental Levels: Focus on Mitochondrial Function and Larval Activity in Early Staged Zebrafish (*Danio rerio*). Toxicology.

[7] Woodward E.E., Hladik M.L., Main A.R., Cahn M., Orlando J.L., Teerlink J. (2022). Comparing Imidacloprid, Clothianidin, and Azoxystrobin Runoff from Lettuce Fields Using a Soil Drench or Treated Seeds in the Salinas Valley, California. Environ. Pollut..

[8] Esser L., Yu C.-A., Xia D. (2014). Structural Basis of Resistance to Anti-Cytochrome Bc1 Complex Inhibitors: Implication for Drug Improvement. Curr. Pharm. Des..

[9] Cui F., Chai T., Liu X., Wang C. (2017). Toxicity of Three Strobilurins (Kresoxim-Methyl, Pyraclostrobin, and Trifloxystrobin) on Daphnia Magna. Environ. Toxicol. Chem..

[10] Wang S., Wang J., Zhang X., Xu X.T., Wen Y., He J., Zhao Y.H. (2021). Freshwater Quality Criteria of Four Strobilurin Fungicides: Interspecies Correlation and Toxic Mechanism. Chemosphere.

[11] Li H., Cao F., Zhao F., Yang Y., Teng M., Wang C., Qiu L. (2018). Developmental Toxicity, Oxidative Stress and Immunotoxicity Induced by Three Strobilurins (Pyraclostrobin, Trifloxystrobin and Picoxystrobin) in Zebrafish Embryos. Chemosphere.

[12] Ho E., Galougahi K.K., Liu C.-C., Bhindi R., Figtree G.A. (2013). Biological Markers of Oxidative Stress: Applications to Cardiovascular Research and Practice. Redox Biol..

[13] Al-Eryani L., Wahlang B., Falkner K.C., Guardiola J.J., Clair H.B., Prough R.A., Cave M. (2015). Identification of Environmental Chemicals Associated with the Development of Toxicant-Associated Fatty Liver Disease in Rodents. Toxicol. Pathol..

[14] Cao F., Li H., Zhao F., Wu P., Qian L., Huang L., Pang S., Martyniuk C.J., Qiu L. (2019). Parental Exposure to Azoxystrobin Causes Developmental Effects and Disrupts Gene Expression in F1 Embryonic Zebrafish (*Danio rerio*). Sci. Total Environ..

[15] Cao F., Wu P., Huang L., Li H., Qian L., Pang S., Qiu L. (2018). Short-Term Developmental Effects and Potential Mechanisms of Azoxystrobin in Larval and Adult Zebrafish (*Danio rerio*). Aquat. Toxicol..

[16] Simon J.M., Paranjape S.R., Wolter J.M., Salazar G., Zylka M.J. (2019). High-Throughput Screening and Classification of Chemicals and Their Effects on Neuronal Gene Expression Using RASL-Seq. Sci. Rep..

[17] Serim I., Demirel H.H., Zemheri-Navruz F., Ince S. (2024). Taurine Exhibits Antioxidant, Anti-Inflammatory, and Antiapoptotic Effects against Pyraclostrobin Exposure in Rats. Toxicol. Res..

[18] Vodkin I., Valasek M.A., Bettencourt R., Cachay E., Loomba R. (2015). Clinical, Biochemical and Histological Differences between HIV-Associated NAFLD and Primary NAFLD: A Case-Control Study. Aliment. Pharmacol. Ther..

[19] Foulds C.E., Trevino L.S., York B., Walker C.L. (2017). Endocrine-Disrupting Chemicals and Fatty Liver Disease. Nat. Rev. Endocrinol..

[20] Heindel J.J., Blumberg B., Cave M., Machtinger R., Mantovani A., Mendez M.A., Nadal A., Palanza P., Panzica G., Sargis R. (2017). Metabolism Disrupting Chemicals and Metabolic Disorders. Reprod. Toxicol..

[21] Younossi Z.M., Koenig A.B., Abdelatif D., Fazel Y., Henry L., Wymer M. (2016). Global Epidemiology of Nonalcoholic Fatty Liver Disease-Meta-Analytic Assessment of Prevalence, Incidence, and Outcomes. Hepatology.

[22] Estes C., Anstee Q.M., Teresa Arias-Loste M., Bantel H., Bellentani S., Caballeria J., Colombo M., Craxi A., Crespo J., Day C.P. (2018). Modeling NAFLD Disease Burden in China, France, Germany, Italy, Japan, Spain, United Kingdom, and United States for the Period 2016–2030. J. Hepatol..

[23] Morrow J.D., Hill K.E., Burk R.F., Nammour T.M., Badr K.F., Roberts L.J. (1990). A Series of Prostaglandin F2-like Compounds Are Produced in Vivo in Humans by a Non-Cyclooxygenase, Free Radical-Catalyzed Mechanism. Proc. Natl. Acad. Sci. USA.

[24] Roberts L.J., Morrow J.D. (2000). Measurement of F2-Isoprostanes as an Index of Oxidative Stress in Vivo. Free Radic. Biol. Med..

[25] Valavanidis A., Vlachogianni T., Fiotakis C. (2009). 8-Hydroxy-2′ -Deoxyguanosine (8-OHdG): A Critical Biomarker of Oxidative Stress and Carcinogenesis. J. Environ. Sci. Health Part C Environ. Carcinog. Ecotoxicol. Rev..

[26] Qin R., Zhang B., Zhu H., Chen Y., Song S., Zhang T. (2024). Exposure to Per- and Polyfluoroalkyl Substances, Neonicotinoid Insecticides, Benzotriazoles and Benzothiazoles: Associations with Human Non-Alcoholic Fatty Liver Disease. Environ. Chem. Ecotoxicol..

[27] Song S., Gao Y., Feng S., Cheng Z., Huang H., Xue J., Zhang T., Sun H. (2024). Widespread Occurrence of Two Typical N, N’-Substituted p-Phenylenediamines and Their Quinones in Humans: Association with Oxidative Stress and Liver Damage. J. Hazard. Mater..

[28] Wang X., Xie Q. (2022). Secondary Factors and Diagnosis of Nonalcoholic Fatty Liver Disease. Chin. Hepatol..

[29] Li Y.M., Fan J.G. (2019). Guidelines of Prevention and Treatment for Alcoholic Liver Disease (2018, China). J. Dig. Dis..

[30] Bai X., Zhang B., He Y., Hong D., Song S., Huang Y., Zhang T. (2020). Triclosan and Triclocarbon in Maternal-Fetal Serum, Urine, and Amniotic Fluid Samples and Their Implication for Prenatal Exposure. Environ. Pollut..

[31] Zhang B., Zhang H., Bai X., Zhang T., Xue J., Lu S., Kannan K. (2022). Placental Transfer of Bisphenol Diglycidyl Ethers (BDGEs) and Its Association with Maternal Health in a Population in South of China. Eco-Environ. Health.

[32] Rodrigues E.T., Lopes I., Pardal M.A. (2013). Occurrence, Fate and Effects of Azoxystrobin in Aquatic Ecosystems: A Review. Environ. Int..

[33] Liu J., Xia W., Wan Y., Xu S. (2021). Azole and Strobilurin Fungicides in Source, Treated, and Tap Water from Wuhan, Central China: Assessment of Human Exposure Potential. Sci. Total Environ..

[34] Hu W., Hsiao Y.-C., Morrison-Welch N., Lamberti S., Liu C.-W., Lin W., Engel S.M., Lu K., Zylka M.J. (2024). Co-Detection of Azoxystrobin and Thiabendazole Fungicides in Mold and Mildew Resistant Wallboards and in Children. Heliyon.

[35] Liu J., Wan Y., Jiang Y., Xia W., He Z., Xu S. (2022). Occurrence of Azole and Strobilurin Fungicides in Indoor Dust from Three Cities of China. Environ. Pollut..

[36] Bo H., Wang J., Guo C., Qin R., Lu X. (2008). Determination of Strobilurin Fungicide Residues in Food by Gas Chromatography-Mass Spectrometry. Chin. J. Anal. Chem..

[37] Kumar Y.B., Shabeer T.P.A., Jadhav M., Banerjee K., Hingmire S., Saha S., Rai A.B. (2020). Analytical Method Validation, Dissipation and Safety Evaluation of Combination Fungicides Fenamidone plus Mancozeb and Iprovalicarb plus Propineb in/on Tomato. J. Food Sci. Technol.-Mysore.

[38] Zhang H., Feng S., Song S., Zhao Q., Gao Y., Zhang T. (2025). First Evidence in the Association of Phenolic Endocrine-Disrupting Chemicals with Secondary Non-Alcoholic Fatty Liver Disease: A Case-Control Study in South China. Environ. Pollut..

[39] Mogahed E., Sayed A., Khalifa S., El-Hennawy A., El-Raziky M. (2020). Causes of Secondary Non-Alcoholic Fatty Liver Disease in Non-Obese Children below 10 Years. Eur. J. Pediatr..

[40] Hu W., Liu C.-W., Jimenez J.A., McCoy E.S., Hsiao Y.-C., Lin W., Engel S.M., Lu K., Zylka M.J. (2022). Detection of Azoxystrobin Fungicide and Metabolite Azoxystrobin-Acid in Pregnant Women and Children, Estimation of Daily Intake, and Evaluation of Placental and Lactational Transfer in Mice. Environ. Health Perspect..

[41] Bi Y., Han L., Qin F., Song S., Lv X., Dong Q., Qiao C., Ren B. (2022). Method Validation, Residue Analysis and Dietary Risk Assessment of Trifloxystrobin and Trifloxystrobin Acid in Milk, Eggs and Pork. Biomed. Chromatogr..

[42] Arena M., Auteri D., Barmaz S., Bellisai G., Brancato A., Brocca D., Bura L., Byers H., Chiusolo A., Marques D.C. (2017). Peer Review of the Pesticide Risk Assessment of the Active Substance Trifloxystrobin. EFSA J..

[43] European Food Safety Authority (2010). Conclusion on the Peer Review of the Pesticide Risk Assessment of the Active Substance Azoxystrobin. https://www.efsa.europa.eu/en/efsajournal/pub/1542.

[44] European Food Safety Authority (2007). Conclusion Regarding the Peer Review of the Pesticide Risk Assessment of the Active Substance Fluoxastrobin. https://www.efsa.europa.eu/en/efsajournal/pub/rn-102.

[45] Martinez-Moral M.-P., Kannan K. (2019). How Stable Is Oxidative Stress Level? An Observational Study of Intra- and Inter-Individual Variability in Urinary Oxidative Stress Biomarkers of DNA, Proteins, and Lipids in Healthy Individuals. Environ. Int..

[46] Kovacevic M., Stjepanovic N., Hackenberger D.K., Loncaric Z., Hackenberger B.K. (2022). Comprehensive Study of the Effects of Strobilurin-Based Fungicide Formulations on Enchytraeus Albidus. Ecotoxicology.

[47] Liu X., Wang Y., Chen H., Zhang J., Wang C., Li X., Pang S. (2018). Acute Toxicity and Associated Mechanisms of Four Strobilurins in Algae. Environ. Toxicol. Pharmacol..

[48] Zemheri-Navruz F., Ince S., Arslan-Acaroz D., Acaroz U., Demirel H.H., Demirkapi E.N. (2023). Resveratrol Alleviates Pyraclostrobin-Induced Lipid Peroxidation, Oxidative Stress, and DNA Damage in Rats. Environ. Sci. Pollut. Res. Int..

[49] Gollan J.L., Zucker S.D. (1996). A New Voyage of Discovery: Transport through the Hepatocyte. Trans. Am. Clin. Climatol. Assoc..

[50] Gan C., Yuan Y., Shen H., Gao J., Kong X., Che Z., Guo Y., Wang H., Dong E., Xiao J. (2025). Liver Diseases: Epidemiology, Causes, Trends and Predictions. SIGNAL Transduct. Target. Ther..

[51] El-Hak H.N.G., Al-Eisa R.A., Ryad L., Halawa E., El-Shenawy N.S. (2022). Mechanisms and Histopathological Impacts of Acetamiprid and Azoxystrobin in Male Rats. Environ. Sci. Pollut. Res..

[52] Han Y., Liu T., Wang J., Wang J., Zhang C., Zhu L. (2016). Genotoxicity and Oxidative Stress Induced by the Fungicide Azoxystrobin in Zebrafish (*Danio rerio*) Livers. Pestic. Biochem. Physiol..

[53] Huang T., Souders C.L., Wang S., Ganter J., He J., Zhao Y.H., Cheng H., Martyniuk C.J. (2021). Behavioral and Developmental Toxicity Assessment of the Strobilurin Fungicide Fenamidone in Zebrafish Embryos/Larvae (*Danio rerio*). Ecotoxicol. Environ. Saf..

[54] Uckun A.A., Oz O.B. (2021). Evaluation of the Acute Toxic Effect of Azoxystrobin on Non-Target Crayfish (Astacus leptodactylusEschscholtz, 1823) by Using Oxidative Stress Enzymes, ATPases and Cholinesterase as Biomarkers. Drug Chem. Toxicol..

[55] Usman M., Bakhtawar N. (2020). Vitamin E as an Adjuvant Treatment for Non-Alcoholic Fatty Liver Disease in Adults: A Systematic Review of Randomized Controlled Trials. Cureus J. Med. Sci..

