# Supplementary material for: First Human Biomonitoring Evidence of Strobilurin Fungicide Exposure in South China: Impact on Oxidative Stress and Liver Damage
Source: Toxics. 2025 Oct 23;13(11):908. doi: 10.3390/toxics13110908 (PMC12656519; doi:10.3390/toxics13110908)
Supplement: Supplementary file 1 [file toxics-13-00908-s001.zip › toxics-3903194-supplementary.pdf]

Supporting Information

First human biomonitoring evidence of strobilurin fungicides exposure in South China: Impact on oxidative stress and liver damage

Bo Zhang[1, 2], Shuai Feng[2], Yanxia Gao[2], Wenxi Xie[3], Yiyu Chen[2], Shiming Song[4*]

[1]Key Laboratory of Guangdong Higher Education Institutions of Northeast Guangdong New Functional Materials，School of Chemistry and Environment, Jiaying University, Meizhou, 514015, China

[2]School of Environmental Science and Engineering, Sun Yat-Sen University, Guangzhou 510275, China

[3]Qiqihar Environmental Monitoring Station, Qiqihar 161005, China

[4]School of Agriculture and Biotechnology, Sun Yat-Sen University, Shenzhen 518107, China

Corresponding Author

*Shiming Song

School of Agriculture and Biotechnology, Sun Yat-Sen University

66 Gongchang Road, Shenzhen, 518107, China

Tel: 86-0755-23260257

E-mail: songshm3@mail.sysu.edu.cn

Submission to: *Toxics*

Sample Extraction and Instrumental Analysis.

Concentrations of target antimicrobials in serum samples were quantified by reference standardization. Specifically, 0.5 mL serum were defrosted in 4 °C and transferred into a 15 mL PP-tube. ISs solution was spiked into serum samples with 1.0 ng (5.0 ng/mL in serum) and then added 40 μL β-glucuronidase and sulfatase in sodium acetate buffer that contained 82 units. The samples were incubated at 37 °C for a duration of 12 h and then extracted three times with 2.0 mL ethyl acetate. This step hydrolyzes glucuronide conjugates, allowing for the measurement of total F_2-isoprostanes, which is considered the gold standard approach for assessing systemic oxidative stress burden *in vivo*. For each extraction, the samples were shaken for 0.5 h at 300 r/min and subsequently centrifugated for 5 min at 8000 rpm. The combined supernatants were transferred into a new 15-mL PP tube and gently evaporated under a stream of nitrogen gas until near dryness. The residuals were finally redissolved with 0.2 mL methanol and centrifugated for 15 min at 12000 rpm, then the supernatant was transferred into a 2 mL-vial for instrumental analysis.

Target analytes were analyzed using an Agilent 1290 Infinity II high-performance liquid chromatography system (Agilent Technologies, Santa Clara, CA), coupled with a SCIEX 5500 triple quadrupole mass spectrometer (SCIEX, Framing-ham, MA). This system was equipped with a Zorbax SB-C18 column (100 mm × 2.1 mm, 3.5 μm; Agilent) and operated at room temperature. The injection volume was 2 μL and the flow rate was 0.3 mL/min. SFs were analyzed in electrospray ionization positive mode (ESI^+). The mobile phases employed in the analysis consisted of a 2 mM ammonium acetate, as well as a mixture of Methanol and acetonitrile (1/1, v/v) for ESI^-, methanol and water containing 0.1% formic acid for ESI^+ as mobile phases A and B, respectively. Quantification of the targeted analytes was performed using the multiple reaction monitoring (MRM) mode. The operation parameters for the ESI source were as follows: source temperature of 500 °C; ion spray voltage of -4500 V (ESI^-) and 5000V (ESI^+); column temperature of 25 °C; GSI, GSII and CUR were set at 40, 40 and 35 psi, respectively. Detailed information on the analytical method was

shown in **Table S1**, and the HPLC and MS/MS parameters were presented in **Table S2**.

Table S1. The gradient of mobile phases for SFs

Time(min)	A (%)	B (%)
0.0	95	5
2.0	85	15
5	40	60
7	20	80
10	1	99
11	1	99
11.5	95	5
14	95	5

Table S2. The precursor (Q1), product ion (Q3), declustering potential (DP) and collision energy (CE) for targeted antimicrobials and oxidative stress indicators quantification and qualification.

Analytes	Q1(m/z)	Q3 (m/z)	DP[a]	CE[b]
AZ	404	372	81	22
AZ-d_4	408	348	30	35
FE	312	236	151	21
FLUO	459	427	56	25
ORY	392	205	43	21
PICO	368	145	89	32
TRIF	409	186	131	29
AZ-ACID	390	329	50	41
TRIF-ACID	395	186	63	22
8-iso-$PGF_{2\alpha}$	353	193.5	-42	-36
11-iso-$PGF_{2\alpha}$	353	193	-82	-33
15-iso-$PGF_{2\alpha}$	353	193.1	-40	-35
D_4-8-iso-$PGF_{2\alpha}$	357	197	-103	-36
8-OHdG	284	168	50	25
$^{13}C_{12}$-8-OHdG	287	171	41	17

[a] DP represents declustering potential; [b] CE represents collision enery

Table S3. Associations [a] between serum concentration of SF (DF >50%) and selected liver function indictors

	AST	GGT
Outcomes	β (95% CI)	β (95% CI)
AZ	0.003 (-0.060, 0.066)	-0.023 (-0.115, 0.070)
FE	0.032 (-0.036, 0.100)	-0.011 (-0.117, 0.095)
FLUO	0.040 (-0.032, 0.112)	-0.024 (-0.121, 0.073)

[a] Estimates were calculated by using the linear regression model, the concentration below LOQ was excluded from this calculation, and the concentrations of all SF were lg-transformed for estimation.

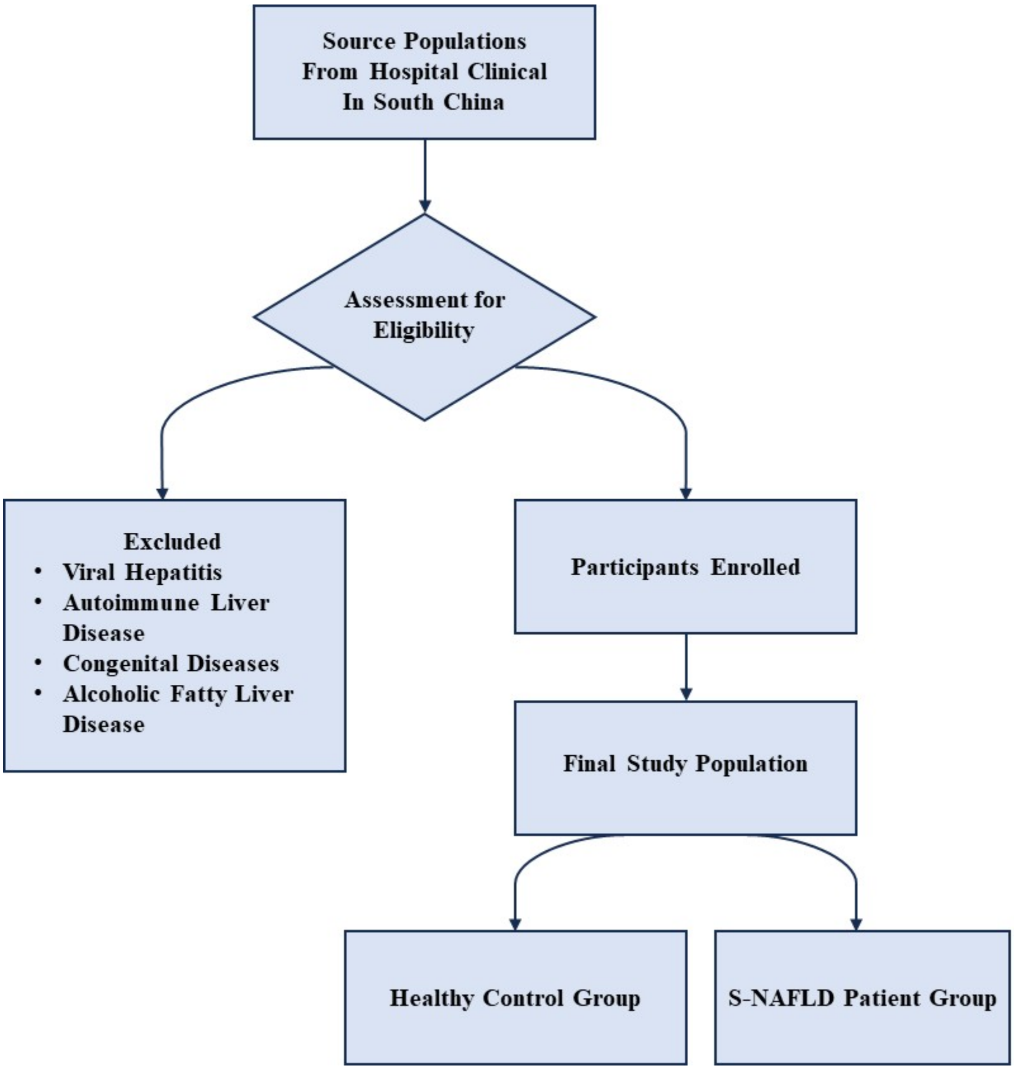

Fig. S1. Schematic diagram of the analytical cohort structure.

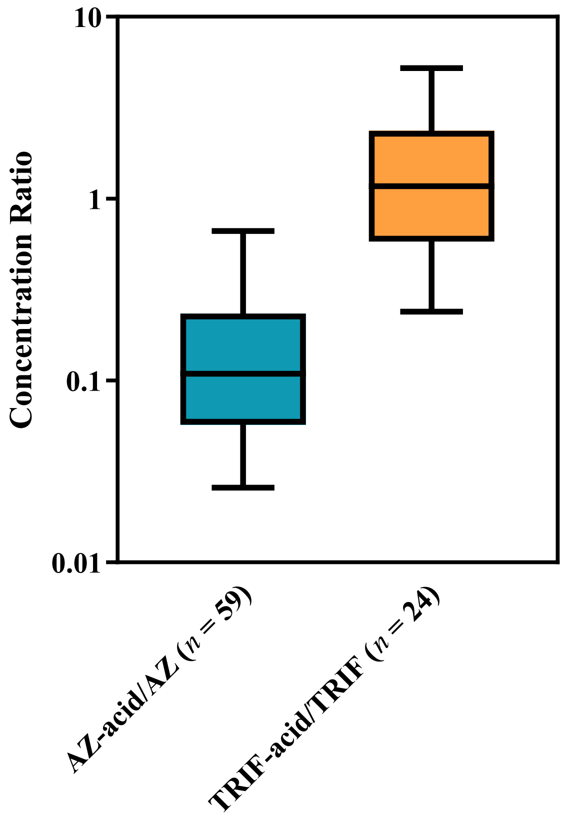

Fig. S2. Concentration ratio of AZ/AZ-acid and TRIF/TRIF-Acid in serum samples collected from all participants. *Participants having both detectable parent compounds and metabolites were included from this calculation.*
